# Potentiometric Biosensing of Ascorbic Acid, Uric Acid, and Cysteine in Microliter Volumes Using Miniaturized Nanoporous Gold Electrodes

**DOI:** 10.3390/bios11010010

**Published:** 2020-12-28

**Authors:** Christopher J. Freeman, Borkat Ullah, Md. Shafiul Islam, Maryanne M. Collinson

**Affiliations:** 1 Department of Chemistry and Biochemistry, Old Dominion University, Norfolk, VA 23529, USA; cjfreema@odu.edu; 2 Department of Chemistry, Virginia Commonwealth University, Richmond, VA 23284, USA; ullahb@mymail.vcu.edu (B.U.); islamm3@mymail.vcu.edu (M.S.I.)

**Keywords:** potentiometry, oxidation reduction potential (ORP), open-circuit potential, redox potential, Nernst equation

## Abstract

Potentiometric redox sensing is a relatively inexpensive and passive approach to evaluate the overall redox state of complex biological and environmental solutions. The ability to make such measurements in ultra-small volumes using high surface area, nanoporous electrodes is of particular importance as such electrodes can improve the rates of electron transfer and reduce the effects of biofouling on the electrochemical signal. This work focuses on the fabrication of miniaturized nanoporous gold (NPG) electrodes with a high surface area and a small footprint for the potentiometric redox sensing of three biologically relevant redox molecules (ascorbic acid, uric acid, and cysteine) in microliter volumes. The NPG electrodes were inexpensively made by attaching a nanoporous gold leaf prepared by dealloying 12K gold in nitric acid to a modified glass capillary (1.5 mm id) and establishing an electrode connection with copper tape. The surface area of the electrodes was ~1.5 cm^2^, providing a roughness factor of ~16 relative to the geometric area of 0.09 cm^2^. Scanning electron microscopy confirmed the nanoporous framework. A linear dependence between the open-circuit potential (OCP) and the logarithm of concentration (e.g., Nernstian-like behavior) was obtained for all three redox molecules in 100 μL buffered solutions. As a first step towards understanding a real system, the response associated with changing the concentration of one redox species in the presence of the other two was examined. These results show that at NPG, the redox potential of a solution containing biologically relevant concentrations of ascorbic acid, uric acid, and cysteine is strongly influenced by ascorbic acid. Such information is important for the measurement of redox potentials in complex biological solutions.

## 1. Introduction

Potentiometry is an important electroanalytical technique routinely used to measure the concentration (activity) of small ions (e.g., H^+^, K^+^, F^−^) in solution [1]. In contrast to other electrochemical sensing techniques such as chronoamperometry, differential pulse voltammetry (DPV), or cyclic voltammetry (CV), no current flows. As a result, there is no perturbation to the interface, and no changes in the chemical composition of the sample take place. It is a quick and passive measurement technique that requires minimum instrumentation and can be easily miniaturized for field portability [1,2,3,4,5,6]. Billions of potentiometric measurements are likely made each year using traditional membrane-based ion-selective electrodes.

Redox potentiometry using metallic redox electrodes, albeit less studied, has also been shown to be a useful tool to evaluate the redox properties of complex environmental or biological samples and determine the concentration of small molecules [1,7,8,9]. In this experiment, the open-circuit potential (OCP) or zero-current potential of an indicating, redox electrode (E_Ind_) (i.e., inert electrode such as gold or platinum) is measured with respect to a reference (E_ref_) electrode using a high-impedance voltmeter, such that E_measured_ = E_Ind_ − E_ref_. For E_measured_ to reflect what takes place at the indicator electrode, the potential of the reference must remain constant. Unlike traditional membrane ion-selective electrodes (ISEs) [1,2,3,4,5,6], the redox electrode is not specific to the ion for which it was created but rather can collectively respond to any number of species present in the solution. The measurement is particularly sensitive to the concentration of the redox species present in solution, the rate of electron transfer between the redox species and the electrode, the electrode material (e.g., gold, platinum, etc.), and its surface composition [7,8,9,10]. Because the measured potential is often a mixed potential [11,12], it can make the interpretation and understanding of these types of OCP measurements challenging. Nevertheless, redox potentiometry has been successfully used to evaluate the overall redox state of complex samples. Examples include water and soil samples [7,13,14,15,16,17,18], blood and blood products [19,20,21,22], milk [23], tea fermentation [24], fish [25,26], and cheese making [27]. Under certain conditions, the direct potentiometric determination of small molecules and ions such as hydrogen peroxide [28,29], oxygen [30,31], phosphate [32], and ascorbate [33,34] can be achieved using different metal electrodes and/or polymer coatings to enhance specificity and sensitivity. In more recent work, gold gate field-effect transistor (FET) based sensors for biomolecules have also been reported [35,36]. OCP measurements have also been recently shown to be a promising tool to sensitively detect nanoparticle collision events [37,38,39,40,41], single emulsion droplet collisions at an interface between two immiscible solutions [42,43], and recently enzyme kinetics [44].

While all these studies have demonstrated the promise of redox potentiometry with metallic electrodes, a greater understanding of such measurements and their adaptability to complex biological samples need to be made. Planar platinum electrodes, which are commonly used in redox potentiometry, may not be the optimum electrode to make these measurements due to the propensity for their surfaces to be biofouled [45,46]. Rather, a better choice for redox potentiometry studies in complex biological samples would be an appropriately nanostructured, high surface area electrode known to reduce the effects of biofouling on the electrochemical response as well as improve electron transfer rates of small molecules. While a potentiometric response is typically independent of electrode area [44], electrode areas can indirectly influence the measurement, particularly in complex solutions where the electrode surface is easily fouled or passivated. This passivation can reduce the rate of electron transfer between the electrode and the redox species and result in a loss in sensitivity, most notably at low concentrations [47]. Having plenty of places for electron transfer to take place that is provided by a nanoporous, high surface area electrode, for example, could minimize the impact of biofouling on the potentiometric response. By keeping the footprint of the electrode small, it can be used to measure the redox properties of small sample volumes.

Nanoporous gold (NPG) electrodes prepared by dealloying gold leaf have proven to be promising materials for electrochemical measurements due to their high surface area and ability to increase electron transfer rates for kinetically sluggish reactions [48,49,50]. They have also been shown to be a useful tool to make electrochemical measurements in complex solutions containing known biofouling agents [51,52]. This occurs through a unique biosieving-like mechanism where small redox molecules can enter into the nano-sized pores to exchange electrons while much larger proteins cannot [51]. Nanosized pores are advantageous because they restrict larger biomolecules from entering the framework. However, in some cases, nanopores can be a disadvantage due to mass transport limitations [53] and the correct balance between too big and too small must be made.

While NPG electrodes have been frequently used in amperometric and voltammetric experiments for the detection of small analytes [54,55,56,57,58,59,60], they have been much less studied in redox potentiometry even though they have many promising characteristics. In recent work, they have been used to measure the redox potential of human plasma [47] and red blood cell packets [20]. However, very little is known about what and how many redox species present in these complex solutions influence the measured potential. As a first step toward understanding the potentiometric response in complex solutions and the measurement of mixed potentials, in general, we report on the potentiometric sensing of three small, biologically important redox-active molecules (ascorbic acid, cysteine, and uric acid) as well as a mixture of all three using NPG electrodes. These three antioxidants play an important role in oxidative stress [61], are readily oxidized at metallic electrodes, and are present in plasma and blood. They are believed to be the three major components in blood that could contribute to the measured redox potential; thus, it is important to evaluate their potentiometric behavior individually and then collectively at NPG electrodes.

When working with biological samples, there is a desire to keep the electrode small to minimize the need for large sample sizes. Thus, our experiments begin with the fabrication and validation of a miniature, high surface area nanostructured electrode for redox potentiometric measurements in small volumes of solution. While NPG microelectrodes have been made [62,63,64], these electrodes have a comparably smaller overall electrode area even after taking into account the large roughness factor. The electrodes fabricated herein are ~2 mm in diameter but have a large electrochemically active area that is similar to a standard-sized electrode. These electrodes can be used to measure the redox potential (OCP) in 100 μL volumes of the solution with little effort and inexpensive instrumentation. Because no net current flows in a potentiometric experiment, analyte depletion is not a problem. The proper function of these electrodes in the potentiometric mode was first verified using a poised redox system, potassium ferri/ferrocyanide. Next, the potentiometric response of three biologically important redox molecules (ascorbic acid, uric acid, and cysteine) was evaluated. The three-dimensional cylindrical geometry of the electrodes allowed all measurements to be conducted in a very small volume of solution (100 μL). Nernstian responses were observed for these three redox species. The slope or sensitivity of the calibration curve was ~30 mV/10-fold change in concentration. For the first time, we also describe OCP measurements of a mixed solution containing biologically relevant concentrations of ascorbic acid, uric acid, and cysteine. The results demonstrate that in this mixture of analytes, the experimental OCP is controlled by ascorbic acid, the bioreagent that is more easily oxidized.

## 2. Materials and Methods

Materials: Potassium ferricyanide, potassium phosphate dibasic, L-cysteine (99%), potassium ferrocyanide, potassium phosphate monobasic, hexanes, concentrated nitric acid, potassium chloride, l-ascorbic acid, concentrated sulfuric acid, ethanol (200 proof), uric acid (99%), and (3-mercaptopropyl) trimethoxysilane (95%) were purchased commercially from various vendors (Aldrich (St. Louis, MO, USA), VWR (Radnor, PA, USA), Fisher Scientific (Waltham, MA, USA)) and used as received. Water (18 MΩ/cm) was purified using a Millipore water purification system. All reagent solutions were freshly prepared. Unless otherwise noted, all solutions are prepared in pH 7.4, 0.1 M phosphate buffer solution containing 0.1 M KCl.

NPG electrode fabrication: Glass hematocrit capillary tubes (OD 1.6 mm) were flame sealed and then sonicated for 10 min in ethanol followed by deionized water for 10 min and dried with N_2_ gas. Glass capillary tubes were then O_2_ plasma cleaned using a Southbay PE 2000 plasma etcher at 20 W for 5 min by placing the tubes into a clean glass vial with the desired modification end face-up in the stainless-steel chamber. Cleaned tubes were then soaked with the plasma cleaned side facing down in a 10 mM (3-mercaptopropyl) trimethoxysilane (MPTMS) in hexanes solution for 1 h in a 60 °C water bath. The capillary tubes were then rinsed with hexanes and left to dry. Manetti 12K gold leaf (FineArtStore, a book of 25 loose leaves) was chemically dealloyed in concentrated nitric acid for 13 min and then floated on DI water twice for ~5 min. Conducting copper (Cu) tape (Ted Pella) was attached to the MPTMS-modified tube leaving the functionalized end exposed (~5 mm), which was then used to capture one dealloyed leaf square ensuring the gold contacted both the copper lead and the functionalized glass covering the rounded tip. The dealloyed gold was dried with nitrogen and further cleaned by UV radiation (254 nm, 20 W) for 24 h by placing the electrode face up in a home-built box ~10 cm from the UV source. For testing, NPG electrodes were wrapped securely with Parafilm leaving 1.5 mm of nanoporous gold at the end of the capillary.

Potentiometric Measurements: Unless otherwise noted, open-circuit potential (OCP) measurements were conducted in a two-electrode cell with the NPG electrode and a AgCl-coated Ag wire (reference electrode; 0.1 M KCl) using a CH Instruments 1200A potentiostat or a Metrohm Autolab multichannel potentiostat. A 1.5 mL centrifuge tube cut at the 0.5 mL mark served as the electrochemical cell (Figure S1). The solution volume was 100 μL. A micro flea stir bar was used to quickly stir the solution. To minimize evaporation, the cell was covered with Parafilm. Surface area measurements were undertaken via cyclic voltammetry (CV) in 0.5 M H_2_SO_4_ using a traditional three-electrode cell. The surface morphology of the dealloyed NPG electrodes was examined using a field emission scanning electron microscope (FE-SEM, HITACHI SU-70). X-ray photoelectron spectroscopy (XPS) using a monochromatic Al Kα (1486.68 eV) X-ray source (ThermoFisher ESCA lab 250) with a beam size of 0.5 mm, pass energy of 20 eV, and step size of 0.1 eV was used to determine the percent Ag remaining after dealloying. Samples were cleaned using an Ar plasma to remove any possible contaminants. Data were analyzed using Avantage software and Wagner sensitivity factors.

## 3. Results

### 3.1. Electrode Fabrication

One simple and cost-effective approach to prepare NPG involves chemical dealloying commercially obtained 12 K gold leaf in nitric acid and capturing the dealloyed gold leaf on a conducting substrate (e.g., a gold-coated slide) to make the electrode [54]. However, the gold slides are expensive, millimeters in size, and have limited optical transparency. In addition, mL volumes are needed to make electrochemical measurements with these electrodes. It would be beneficial to have NPG electrodes with a high surface area (similar to a traditional electrode) but a small footprint so that redox potential measurements in small volumes can be easily made.

**Figure 1:**
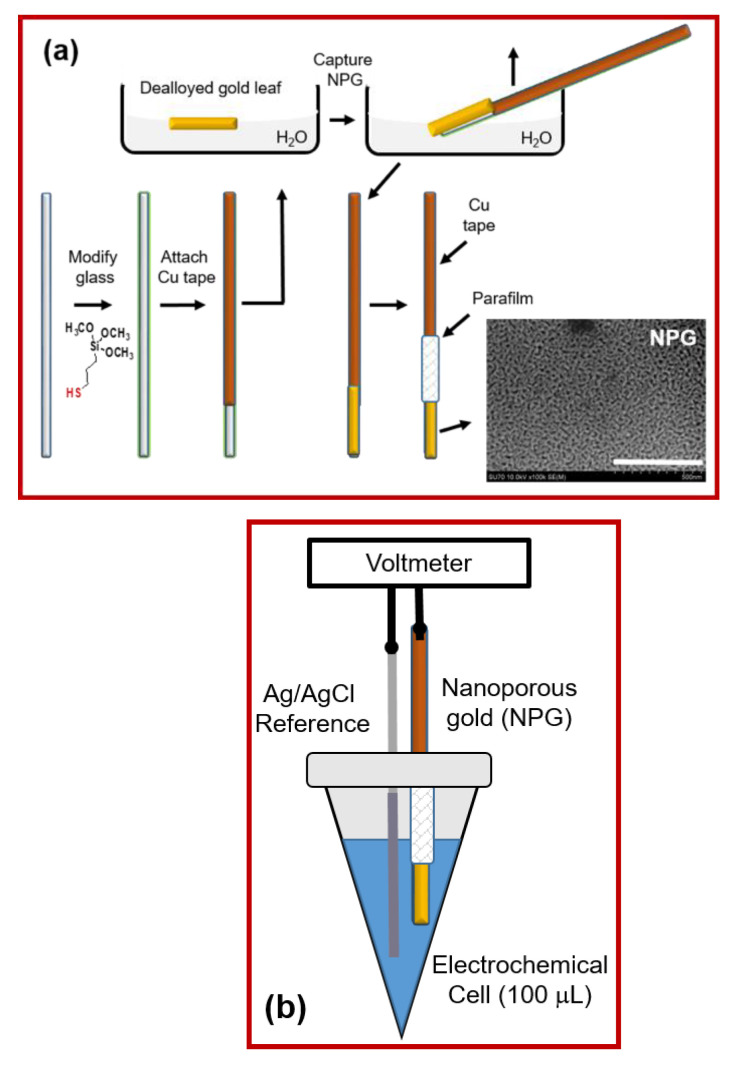
(**a**) Simplified schematic of the fabrication of the miniaturized nanoporous gold electrodes and the corresponding SEM (scale bar is 500 nm). (**b**) Cartoon of the electrochemical cell used to make the potentiometric measurements.

In this report, we describe an approach to decrease the cost as well as reduce the size of the electrodes so that rapid potentiometric measurements in small sample volumes can be made. Such electrodes could be used to measure the redox potential of small volumes of complex chemical systems such as blood products or a collection of electrodes can be used collectively to map heterogeneity in a real sample. With this in mind, we developed a procedure to capture and adhere dealloyed gold leaf on modified glass capillary tubes (1.6 mm OD) using conducting copper tape for the electrical connection. This procedure is depicted in Figure 1a and the electrochemical cell is shown in Figure 1b. A glass capillary tube is first modified with mercaptosilane, which serves as the glue to improve the adhesion of the fragile, dealloyed gold leaf to its surface [65]. To define the geometric area and prevent the solution from contacting the copper tape, Parafilm is stretched and sealed around the capillary tube, or epoxy is used.

A high-resolution SEM image shown in Figure 1a depicts the nanoscale morphology of the gold electrode. Characteristic nanosized pores and ligaments are noted. The pore sizes range from about 10–50 nm. The geometric area of the electrode is 0.09 cm^2^, which is obtained from the diameter of the glass capillary and the length of the exposed gold. The total electrochemically active surface area was obtained by recording a CV in sulfuric acid and measuring the charge associated with the gold oxide peak, Figure S2. Using a conversion factor of 386 μC/cm^2^, the total electrode area is ~16 times larger than the geometric area [54]. The electrodes have 8.56 ± 1.65% Ag (3 different electrodes, 3 points acquired on each electrode) after dealloying as measured by XPS. The XPS spectra for Ag and Au are shown in Figure S3**.** It is known that residual Ag can improve electrocatalytic activity in specific cases [66]. At this time, we do not believe the silver remaining after dealloying influences the OCP when the solutions have relatively high concentrations of redox molecules that exhibit relatively fast kinetics and are not overly surface sensitive. However, variations in the amount of residual silver will likely influence the OCP obtained in buffer as this is an unpoised mixed system and the molecules that likely contribute to the measured potential are surface sensitive. Future experiments will help tease out the importance of residual silver on redox potentiometry experiments.

### 3.2. Potentiometric Response

To verify the newly fabricated NPG electrodes behave in 100 μL solution volumes according to the Nernst equation, a standard oxidation-reduction potential (ORP) calibrant was used: potassium ferricyanide ([Fe(CN)_6_]^3−^) and ferrocyanide ([Fe(CN)_6_]^4−^) in 0.1 M phosphate buffer solution containing 0.1 M KCl [7]. Both the NPG working electrode and Ag/AgCl wire reference electrode were placed in 5 mM [Fe(CN)_6_]^3−^ and the OCP was measured. After a predetermined amount of time for equilibration (typically 100 s), a small aliquot of the reduced form, [Fe(CN)_6_]^4−^ (24 mM), was added to the sample vial while stirring. Immediately after addition, the potential changes to a more negative value and quickly equilibrates. At this point, the solution becomes poised because both forms of the redox couple are present at reasonable concentrations; the resulting potential should be stable and predicted by the Nernst equation. The average value of the OCP was calculated from the last 15 s before the next addition.

**Figure 2:**
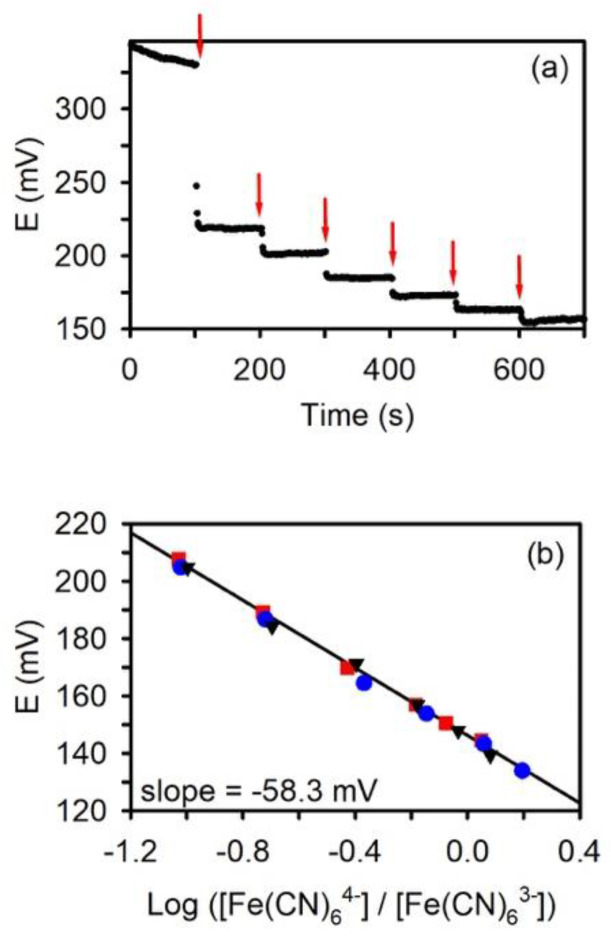
(**a**) OCP (E)-time trace recorded at a nanoporous gold (NPG) electrode following the addition of potassium ferrocyanide (signified by the red arrow) to a potassium ferricyanide receiving solution and (**b**) the corresponding Nernst plot. Each symbol represents a separate experiment with a different NPG electrode. The solid line represents the linear least-squares regression line. y = −58.3 mV + 146.0 mV. R^2^ = 0.9965.

Figure 2a shows the OCP-time trace after successive additions of 2–5 μL aliquots of [Fe(CN)_6_]^4−^ to the [Fe(CN)_6_]^3−^ receiving solution already present in the electrochemical cell. The OCP vs. time trace is very stable, as expected for a poised system. After taking into account dilution factors, the concentration ratio of the reduced to the oxidized form ([Fe(CN)_6_]^4−^/[Fe(CN)_6_]^3−^) was calculated for each addition. The logarithm of the concentration ratio of the two species was used to construct the Nernst plot shown in Figure 2b. The slope was calculated from linear regression (R^2^ = 0.9965) on three data sets (three different electrodes) represented as three different symbols in Figure 2b. The slope was −58.3 ± 0.9 mV with a y-intercept of 146.0 ± 0.5 mV (N = 3). The slope indicates a one-electron transfer system, which is expected for the ferro-ferricyanide redox couple behaving in a Nernstian fashion (−59.2 mV). Because this a poised redox system that follows the Nernst equation, the y-intercept is the formal potential of the redox couple. The y-intercept of 146 mV vs. Ag/AgCl reference electrode in 0.1 M KCl agrees with the expected value of 142 mV for the formal potential of the ferricyanide-ferrocyanide redox couple [7]. We and others have observed similar results for this standard redox system [65,67].

Once the electrodes were validated using a well-behaved redox couple, the response of the electrodes to three biologically relevant redox molecules (ascorbic acid (AA), cysteine (Cys), and uric acid (UA)) was investigated. These systems are more complicated than those obtained for ferri- and ferrocyanide not only because the electrochemistry is more complex but also because these solutions contain only one form of the redox couple. Ascorbic acid is an essential vitamin found in both animal and plant kingdoms and plays an important role in the prevention and treatment of the common cold, mental illness, infertility, cancer, and AIDS [68]. Purine metabolism releases uric acid as a primary end-product and abnormalities in UA can lead to gout, hyperuricemia, and Lesch Nyhan disease [69]. On the other hand, cysteine is an important thiol-containing amino acid and plays a role in protein synthesis as well as in the food, cosmetic, and drug industries [70].

All three of these analytes are also important antioxidants found in blood and blood products and are believed to play a key role in blood redox potential measurements. Most electrochemical experiments on these important bioreagents rely on chronoamperometry or voltammetry, which involves the application of potential and the measurement of current [57,58,59]. However, adsorption and biofouling strongly influence voltammetric measurements while potentiometry is much less affected [67]. Therefore, the potentiometric measurement of AA, UA, and cysteine first in individual solutions and then in mixtures have been studied.

This experiment begins with the addition of small aliquots of either 2.6 mM AA, 4.6 mM Cys, or 9.9 mM UA (all in phosphate buffer) to 100 μL of 0.1 M phosphate buffer (pH = 7.4). The OCP initially measured in the buffer is a mixed potential because no defined redox couples are present in the solution. Upon the addition of a redox molecule, the OCP abruptly changes and establishes a new value. Because only one form of the redox couple is present, the potential cannot be calculated using the Nernst equation and the y-intercept is not equivalent to the redox formal potential. However, the linearity between OCP and the LOG of the concentration is expected providing the pH does not change (a buffer is used in this work) and the concentration of the other form of the redox couple is finite and constant [34]. Equations (1)–(4) demonstrate this relationship for uric acid at pH > pKa:

Urate ion (UA)→Diimine (*DI*) + 1H^+^ + 2e^−^ (1)

(2) $$\;E=E^{o’}+\;\frac{0.059}{2}\;log\frac{\left[DI\right]\;[H^{+}]}{\left[UA\right]}\;$$

(3) $$E=\;E^{o’}+\;\frac{0.059}{2}\log\left[DI\right]+\;\frac{0.059}{2}\log\left[H^{+}\right]-\;\;\frac{0.059}{2}\log\left[UA\right]\;$$

Making the assumptions that the formal potential (*E^o′^*) and the pH stays constant (the solution is buffered) and [*DI*] is small and constant, Equation (3) reduces to the following:

(4) $$E=Constant-\;\frac{0.059}{2}\log\left[UA\right]\;\;\;\;$$

The OCP-time traces are shown in Figure S4 for each of the three biological redox molecules. The first addition results in the largest shift in the OCP because the solution changes from background electrolyte to a dilute solution of that particular redox species. Each successive addition results in a smaller change in the OCP, as expected. In all cases, the OCP becomes more negative as the concentration of the analyte (reductant) increases. The potential reaches a near-constant value as pseudo-equilibrium is reached. Because these molecules have moderately fast electron transfer kinetics at nanoporous gold (see below), the OCP-time traces stabilize once sufficiently high concentrations are present in the solution.

**Figure 3:**
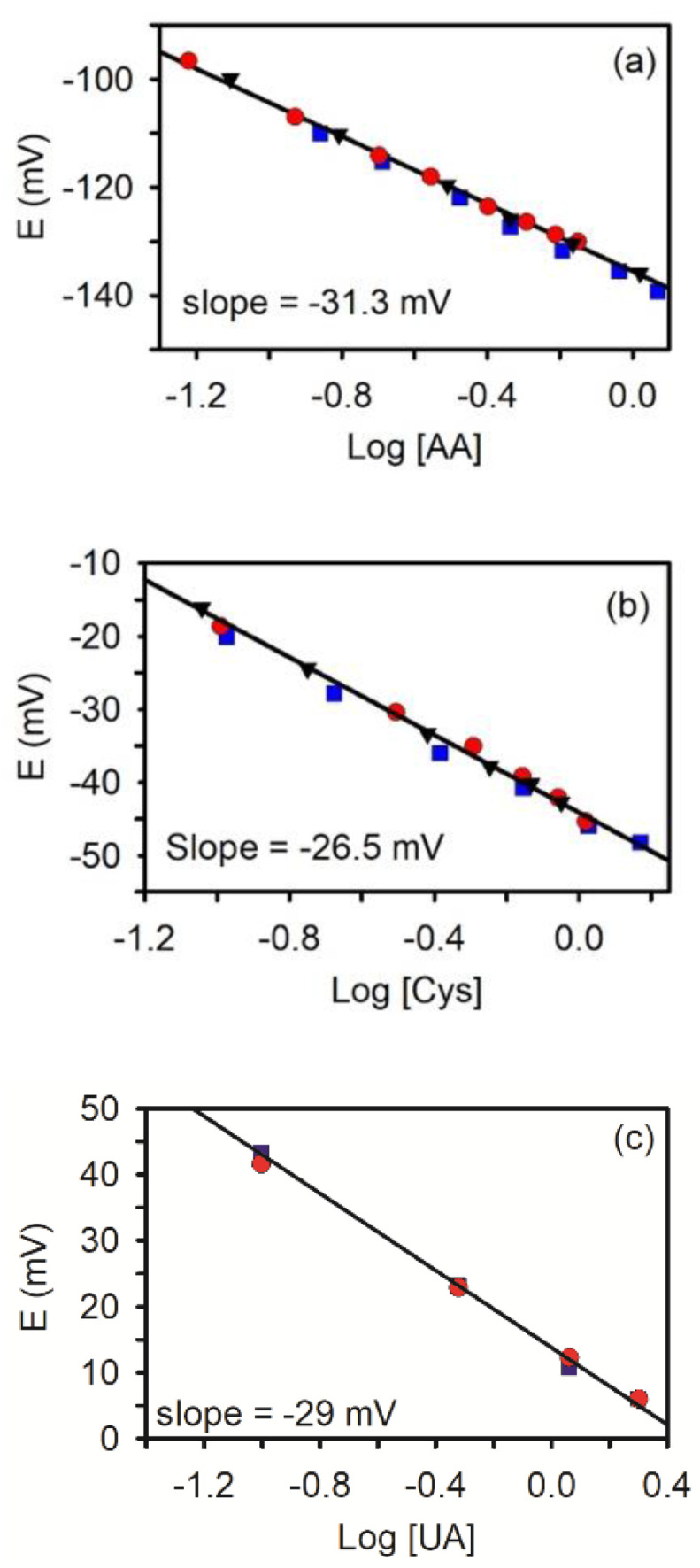
Relationship between the open circuit potential (E) and the logarithm of concentration (in mM) for (**a**) ascorbic acid (AA), (**b**) cysteine (CYS), and (**c**) uric acid (UA). Each symbol represents a separate experiment with a different NPG electrode. The solid line represents the linear least-squares regression line. R^2^ > 0.99.

Figure 3 shows the expected logarithmic dependence of potential on concentration obtained from the OCP-time traces for each of the three redox species. Again, different symbols represent different electrodes and show the reproducibility of the method. It can be noted that the reproducibility between electrodes is very good and all the redox probes show a linear response between potential and logarithm of concentration. The experimental slopes are all consistent with a 2e^−^ process. For ascorbic acid, (Figure 3a), an experimental slope of −31.3 ± 0.4 (N = 3) was obtained over the concentration range of 50 μM to 2 mM; for cysteine (Figure 3b) it was −26.5 ± 0.6 mV (N = 3) over a concentration range of 100 μM to 1.5 mM; for uric acid (Figure 3c), a slope of −29.0 mV (N = 2) was obtained from 100 μM to 2 mM. The slope of these graphs represents the sensitivity of the measurement, which is near the theoretical limit. For ascorbic acid, similar sensitivities were noted using single-walled carbon nanotube carbon fiber electrodes and in microdroplet solutions and solutions containing fibrinogen using NPG [34,65]. The detection limit is estimated to be around 20–50 μM based on when the potential changes from one that is controlled by an added redox molecule to one that is controlled by background processes [38]. Overall the detection sensitivity is similar to other redox potentiometry experiments [34] but not as good as that recently reported using potentiometric FET sensors (e.g., nM for cysteine) [36] or differential pulse voltammetry using graphene/Pt nanocomposite electrodes (0.03–0.15 μM) [60].

### 3.3. Potentiometric Response in a Mixed Solution

When multiple redox species are present in the solution at the same time, the interpretation of OCP becomes more complex. The measured potential depends on many factors including the formal potential, concentration, and electron transfer kinetics of each redox species. Electron transfer rates can also be sensitive to the electrode material [71]. One promising application of OCP measurements involves the potentiometric measurement of the redox potential of a complex solution such as blood or plasma [20]. A shift in the redox potential toward more positive potentials from baseline levels would be indicative of oxidative stress. However, the meaning of the measured potential is complicated because multiple redox species with varying concentrations and formal potentials are present in the solution. As a first step towards understanding a real biological system, we investigated the response of changing the concentration of a redox species, ascorbic acid, in the presence of the other two redox species, cysteine, and uric acid. These three redox species are present in either plasma or blood at levels of 23–85 μM for AA, 50–100 μM for Cys, and 150–470 μM for UA [72].

**Figure 4:**
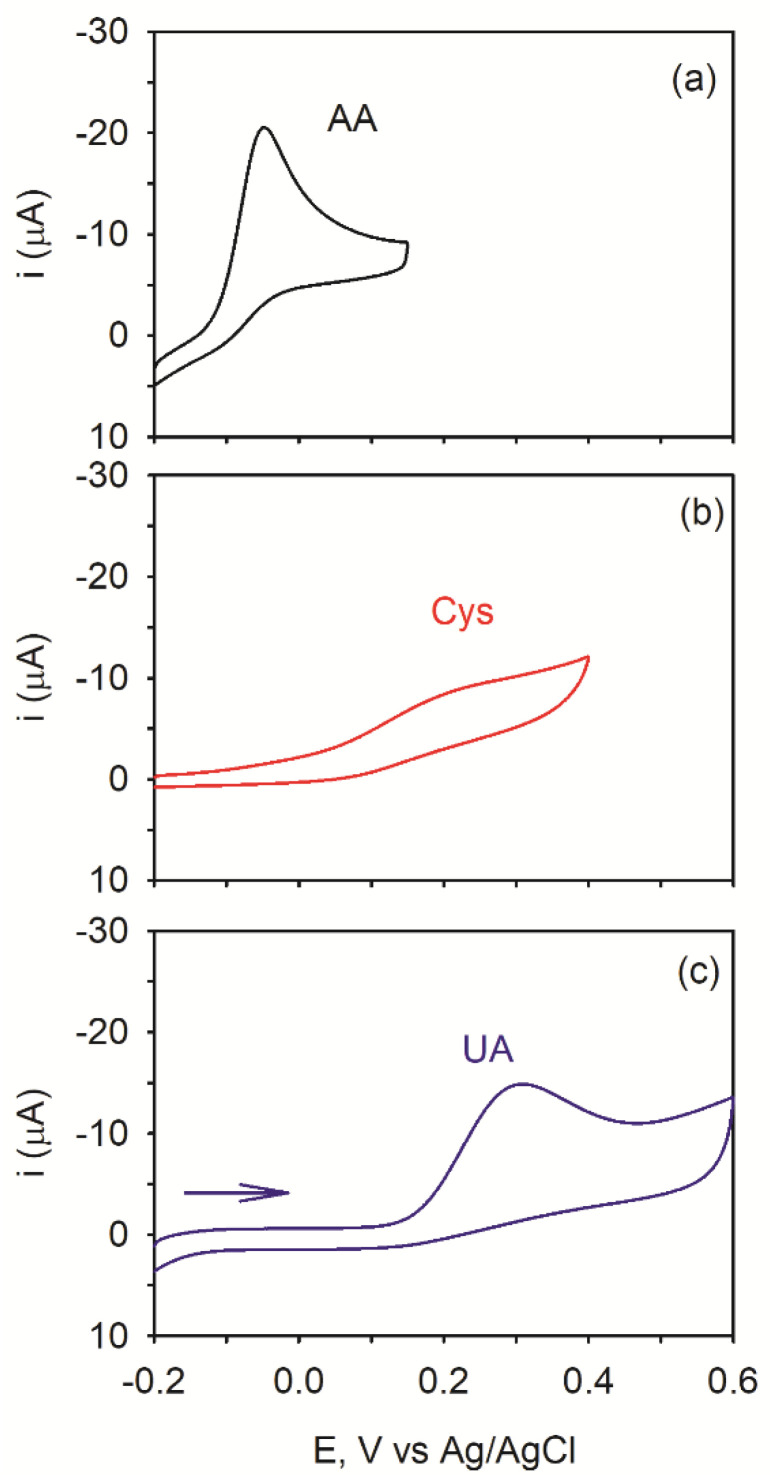
CVs acquired at a separate NPG electrode in a solution containing either (**a**) 0.5 mM ascorbic acid (AA), (**b**) Cysteine (Cys), and (**c**) uric acid (UA) in pH 7.4, 0.1 M PB). Scan rate: 50 mV/s.

In this experiment, cyclic voltammograms (CVs) need to be collected to identify the potential at which each species becomes oxidized. For this to be done, a standard-sized NPG electrode in a standard electrochemical cell must be utilized as these will allow CVs to be collected at slow scan rates without concern for depletion of reagents or radial diffusion. Figure 4 shows the CVs acquired at 50 mV/s at a NPG electrode in a separate solution containing either 0.5 mM AA, UA, or Cys in pH 7.4 phosphate buffer. Separate solutions and separate electrodes were used because all three reagents and/or their oxidation product can adsorb on the electrode [58]. It is evident in these CVs that each redox species can be oxidized at the NPG electrode and have similar electron transfer kinetics. Upon scanning the potential in the positive direction from −0.2 V, AA is oxidized first followed by Cys and then UA.

When all three redox species are present in the same solution, the peak potentials shift to more positive values by ~50 to 100 mV, indicative of adsorption on the electrode surface. It can be noted that ascorbic acid is still the first to be oxidized upon scanning the electrode potential to more positive values followed by cysteine and then uric acid.

**Figure 5:**
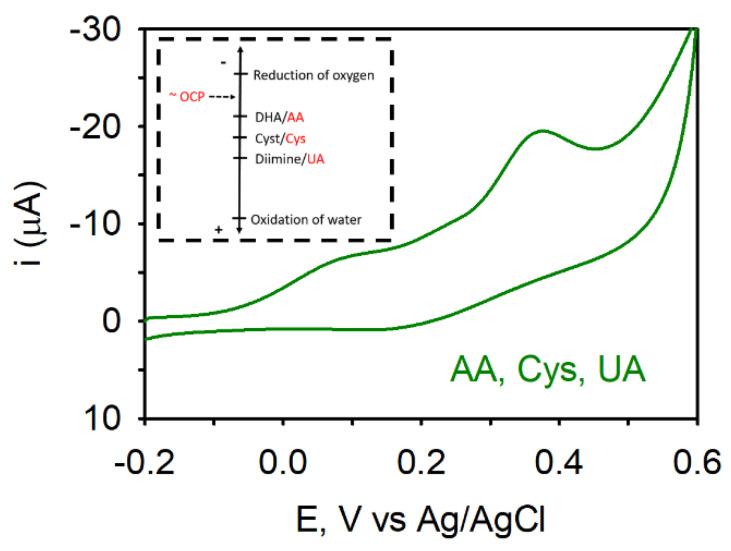
CVs acquired at an NPG electrode in a solution containing 0.5 mM ascorbic acid (AA), cysteine, and uric acid (UA) in pH 7.4 0.1 M PB at a scan rate of 50 mV/s. Inset: Thermodynamic prediction of the approximate location of the OCP (rest) potential of NPG in an equimolar solution containing AA, CYS, and UA.

**Figure 6:**
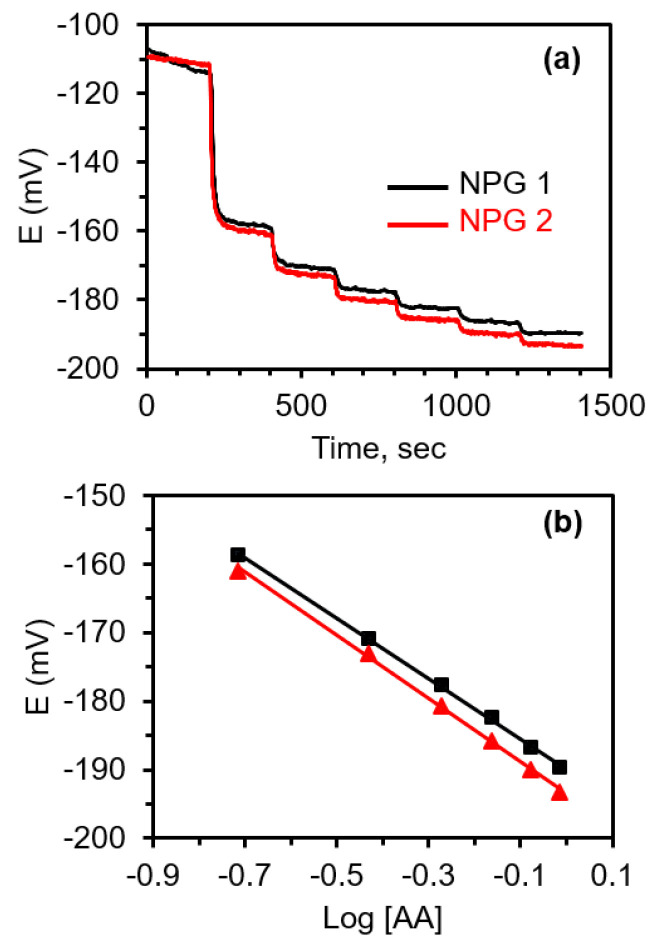
(**a**) OCP (E)—time traces after the addition of ascorbic acid to a buffered solution containing near-physiological concentrations of cysteine (100 μM) and uric acid (300 μM) at an NPG electrode. The results obtained at two different electrodes are shown (black line and red line). With each addition of AA, the OCP becomes more negative. (**b**) The logarithmic dependence of OCP on the concentration of AA (mM). Each symbol represents a different electrode.

Based on thermodynamics alone, the OCP of the mixed solution containing these three redox species is predicted to be negative of the formal potential of AA (Figure 5 inset) [73]. Furthermore, the OCP should be determined by AA at reasonable concentrations. To evaluate this hypothesis, a potentiometric experiment was performed. In this example, AA was added to an initial solution containing both 100 μM Cys and 300 μM UA in 0.1 M pH 7.4 phosphate buffer. These concentrations were chosen because they represent the near-physiological concentrations of Cys and UA in blood. The OCP time-trace for the additions of AA (also prepared in 100 μM Cys and 300 μM UA in phosphate buffer to ensure that the concentration of Cys and UA stay constant) is shown in Figure 6a. The initial OCP starts off being fairly negative of the values typically obtained in phosphate buffer because of the presence of Cys and UA in the receiving solution. The addition of AA to this solution in known increments results in a negative shift in the OCP as expected for the addition of a known reducing agent. With each addition, the OCP becomes more negative. The concentration of AA in the solution after the first addition is 0.19 mM and 0.37 mM after the second addition.

It can be seen in Figure 6b that the OCP changes logarithmically with the concentration of AA. The slope was −43 ± 0.8 mV, which is a little higher than expected but consistent with earlier results for AA [47,65]. When the reverse experiment is done where Cys is added to a solution containing 10 μM AA and 300 μM UA, no clear steps are observed with each subsequent addition of Cys. These results are shown in Figure S5. Rather, the OCP drifts but never really stabilizes. Since the OCP is determined by AA and AA is present in the initial solution, the potential should stay approximately constant at its initial value when small amounts of either UA or Cys are added. In this situation, the potential is ‘poised’ by AA, which is conceptually analogous to the pH of a buffer being set or ‘poised’ by the presence of a conjugate acid/base pair. The addition of a small amount of a base or acid will not change the pH.

Previous experiments observed similar results to those described herein albeit with a different set of analytes and electrode material [34]. These authors showed that the OCP of a SWNT carbon fiber electrode significantly changed when AA was added to an equimolar solution containing four redox active analytes: dopamine, uric acid, serotonin, and dihydroxy-phenyl acetic acid (DOPAC). However, when these redox probes (dopamine, uric acid, serotonin, and DOPAC) were instead added to a PBS solution containing AA, no significant change in the OCP was observed. All these redox molecules have similar electron transfer kinetics and all are also oxidized at potentials more positive of AA. The authors state that the presence of AA decreases the sensitivity of the electrode to the concentration of coexisting neurotransmitters and they explain it in terms of self-driving forces [34]. They also demonstrate how this simple system can be used for in-vivo sensing of AA in live animals [33,34]. The net result of these studies is that the OCP is controlled by AA.

## 4. Conclusions

Redox potential measurements can provide important information regarding the overall redox state of a sample whether it be relatively simple solutions or complex samples such as blood products or sediments. The present work demonstrates that nanoporous gold (NPG) electrodes can successfully respond in a Nernstian-like fashion to biologically important redox species such as AA, UA, and Cys. The NPG electrodes were inexpensively made by starting with white gold leaf and glass capillary tubes. Because of the small size of the electrodes, measurements can be made in sub-milliliter volumes, which bodes well in future testing where only small sample volumes are available. The measurement of the redox potential of a mixed buffered solution containing near-physiological concentrations of AA, UA, and Cys indicates the concentration of AA to be the potential determining factor even when present at a low concentration. These redox molecules have similar kinetics at NPG but are oxidized at more positive potentials than AA.

As compared to amperometric, voltammetric techniques and some potentiometric techniques, the detection limit for these three analytes (in the tens of micromolar range) and the sensitivity (~30 mV per 10-fold change in the concentration) is not as good. However, the strength of this method lies in its simplicity and adaptability to in-field measurements and small volumes. It represents a mediator-free, label-free, and membrane-free approach to the potentiometric sensing of three biologically important redox molecules in 100 μL volumes. Redox potentiometry is particularly useful when the overall redox state of a sample needs to be measured quickly and efficiently with minimal cost using inexpensive instrumentation (e.g., a high impedance voltmeter). This present work is an important step in the understanding of mixed potentials in complex sample solutions containing biologically relevant molecules. It also aids in the understanding of the open-circuit potential in blood and blood products. Our future studies will be aimed at modulating the electrode surface to improve sensitivity and tailoring the selectivity of this redox-based potentiometric sensor for the detection of other biologically important small molecules.

## Data Availability

Data is contained within this article and supplementary material.

## Supplementary Materials

The following are available online at https://www.mdpi.com/2079-6374/11/1/10/s1, Figure S1: Photograph of the electrochemical cell, Figure S2: CV of an NPG electrode in sulfuric acid; Figure S3: XPS spectra; Figure S4: OCP-time traces for the addition of AA, Cys, and UA to a buffer solution; Figure S5: OCP-time traces for the addition of Cys to a solution containing AA and UA.
