# Supplementary material for: Potentiometric Biosensing of Ascorbic Acid, Uric Acid, and Cysteine in Microliter Volumes Using Miniaturized Nanoporous Gold Electrodes
Source: Biosensors (Basel). 2020 Dec 28;11(1):10. doi: 10.3390/bios11010010 (PMC7823660; doi:10.3390/bios11010010)
Supplement: Supplementary file 1 [file biosensors-11-00010-s001.pdf]

biosensors

MDPI

*Article*

# Potentiometric Biosensing of Ascorbic Acid, Uric Acid and Cysteine in Microliter Volumes Using Miniaturized Nanoporous Gold Electrodes

**Christopher J. Freeman [1], Borkat Ullah [2], Md. Shafiul Islam [2] and Maryanne M. Collinson [2,*]**

[1] Department of Chemistry and Biochemistry, Old Dominion University, Norfolk, Virginia, 23529 USA; cjfreema@odu.edu
[2] Department of Chemistry, Virginia Commonwealth University, Richmond, Virginia, 23284 USA;
ullahb@mymail.vcu.edu (B.U.); islamm3@mymail.vcu.edu (M.S.I.)
* Correspondence: mmcollinson@vcu.edu

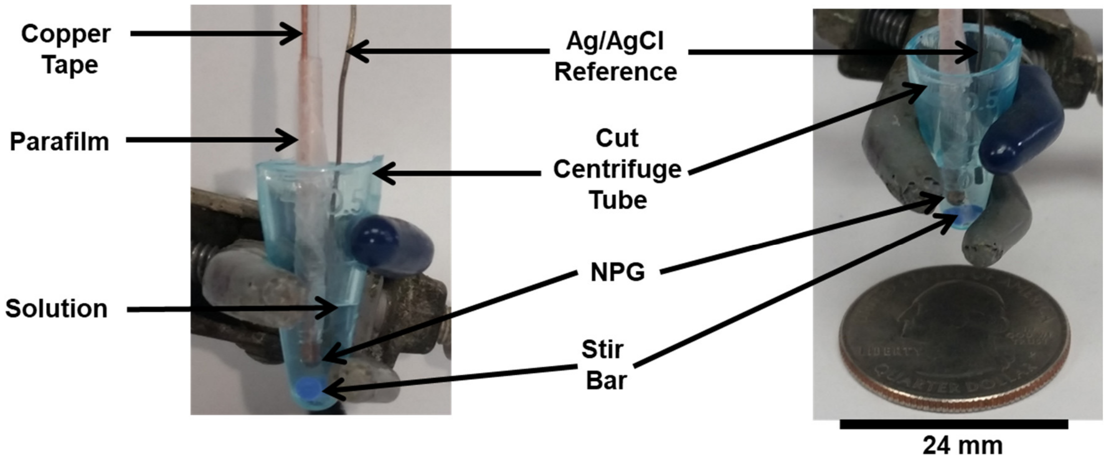


**Figure S1.** Photograph of the electrochemical cell showing a miniature NPG as working electrode and a AgCl-coated Ag wire as the reference electrode in a cut 1.5 mL centrifuge tube. All electrochemical measurements were conducted in a solution volume of 100 µL.

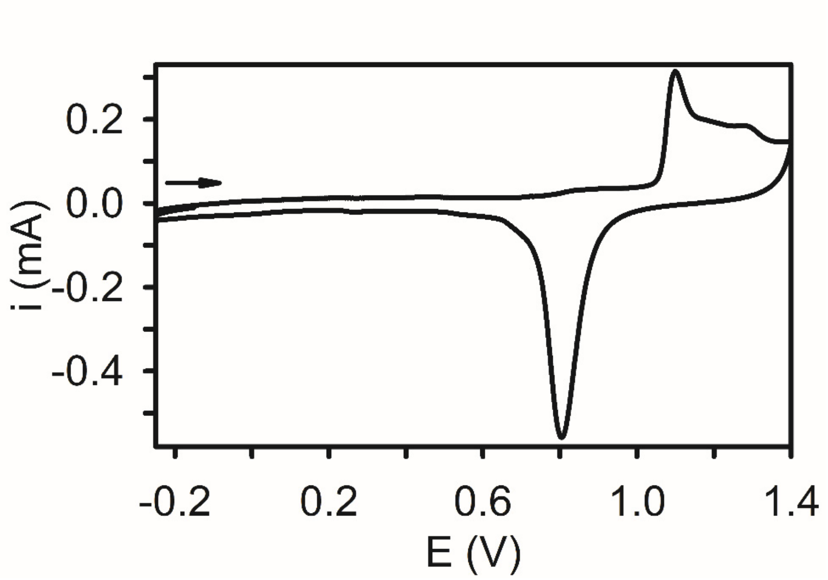


**Figure S2.** Representative cyclic voltammogram of a nanoporous gold electrode in 0.5 M H2SO4 at 100 mV/s. The reference electrode is Ag/AgCl (0.1 M KCl).

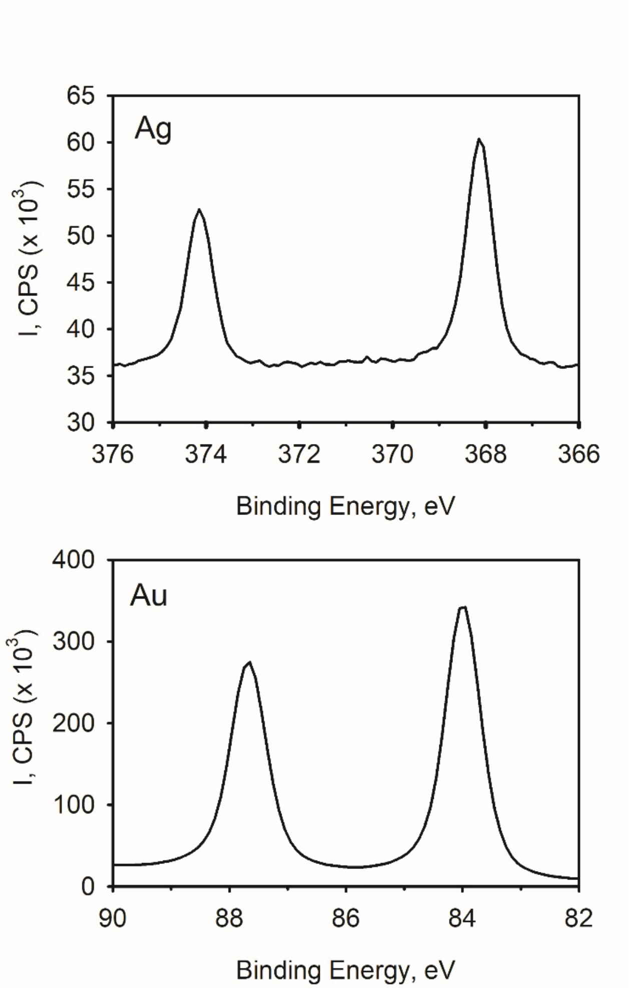


**Figure S3.** XPS spectra for Ag and Au. The binding energies for Ag are 374.14 and 368.13 eV. The binding energies for Au are 87.83 and 84.15 eV. Charge correction was made using C1s at 284.8 eV.

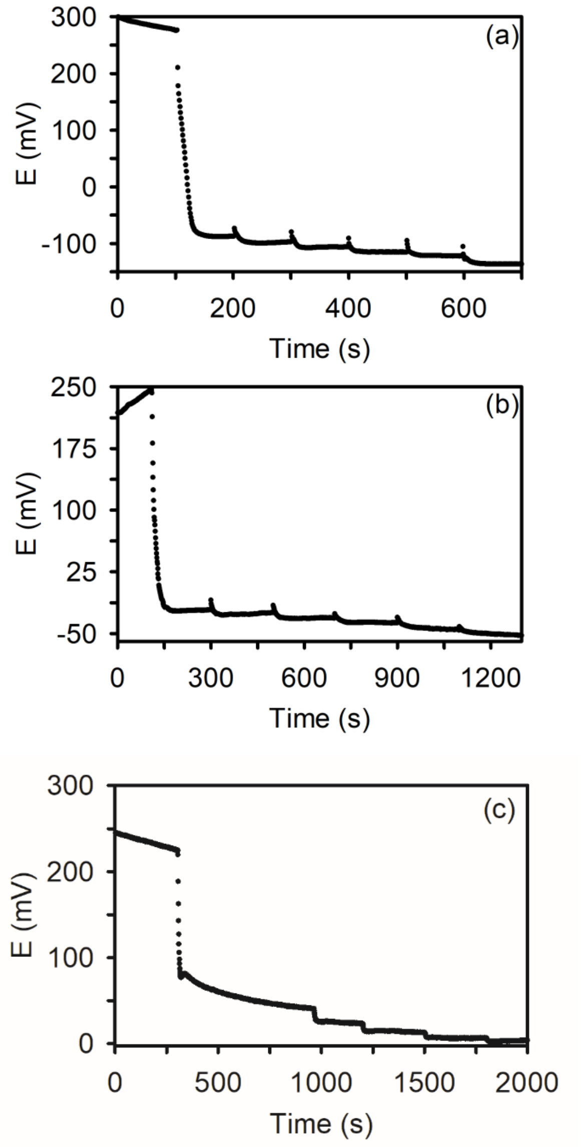


**Figure S4.** Open-circuit potential (OCP)-time traces for the addition of aliquots of (a) ascorbic acid (AA) every 100 seconds, (b) cysteine (Cys) every 200 seconds, and (c) uric acid (UA) every 200 seconds to a 100 µL solution of 0.1 M PBS (0.1 M KCl as electrolyte, pH = 7.4) following an initial wait time of 100 seconds.

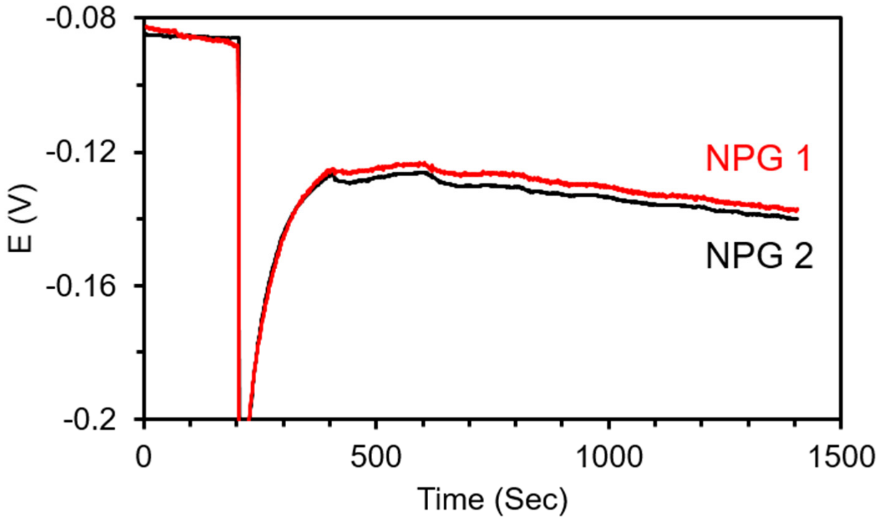


**Figure S5.** Open-circuit potential (OCP)-time traces for the addition of aliquots of cysteine (Cys) (20 mM in 0.1 M PB, pH 7.4, containing AA (10 M) and UA (300 M)) to a receiving solution consisting of pH 7.4 phosphate buffer (0.1 M) containing AA (10 M) and UA (300 M).
